# Water Closets

**Published:** 1874-12

**Authors:** 


### WATER CLOSETS.

It is becoming more and more common to have water closets under the same roof with the family dwelling. When that is the case, there should be at least two in every house; one opening into the rear yard, for servants, with a solid brick wall on three sides, and a door on the fourth, or yard side, hung in such a way as to always swing open, excepting when fastened on the inside by the occupant, thus allowing a constant ventilation.

The other should be on the top floor, in a corner of the building, so that an open window, or, what might be better, a wire screen covering the opening, which would be a sufficient protection against rain and draughts of wind ; and if a similar arrangement could be made overhead, so as to communicate all the time with the out-door air, a good ventilation would be secured. But, in any event, there should be no privy inside a house which does not have a window opening out of doors. This is not practicable in New York dwellings, unless the water closet is at the corner of the house ; and yet there is so little attention paid to this important matter, that we have never been inside of any house in this city which has not the stereotype similarity of a water closet at the head of the stairs, with a window opening into a bed-room, and the door into the hall, leading to the supposition that there is not in all New York an architect who understands his business, or reflects that, in constructing a house, the comfort and health of the inmates should be the very first consideration. Yet it must be said in defence of these gentlemen, that owners of houses have their own views of things, and prefer to build after some previous pattern.

As good arrangements may be noticed at one of the hotels at Chamounix, at the foot of Mont Blanc, openings are left in the wall building inwards, and so high up that no one can close them. But after all the precautions suggested, there will be some perceptible offensive odor. There are many deodorizers, efficient for a longer or shorter time, hence requiring repeated applications, which are apt to be neglected. Any disinfectant which answers the purpose of a continued and automatic application, especially if comparatively inexpensive, should commend itself to every tidy housekeeper. We are now experimenting with a cheap and simple device, by the aid of which substances which disinfect and deodorize, and thus purify the air, may be constantly diffused in cesspools and privies, in sick rooms, and all places wherein bad odors exist, with but slight attention. If it works as well as it promises, it will be a great boon to humanity, and we shall be glad to recommend it to our readers. Meantime, we would remark that a pound or two of chloride of lime, enclosed in a gauze bag, and suspended in places to be disinfected, will absorb and neutralize bad odors and dangerous gases, and may be often renewed at trifling cost.

